# More than just ticking a box…how patient and public involvement improved the research design and funding application for a project to evaluate a cycling intervention for hip osteoarthritis

**DOI:** 10.1186/s40900-015-0013-8

**Published:** 2015-11-27

**Authors:** Lisa M. Andrews, Helen Allen, Zoë A. Sheppard, Guy Baylis, Thomas W. Wainwright

**Affiliations:** 1 Bournemouth University Clinical Research Unit, Bournemouth University, Faculty of Health and Social Sciences, Royal London House, Bournemouth, BH1 3LT UK; 2 Research Design Service South West, c/o Bournemouth University Clinical Research Unit, Bournemouth University, Faculty of Health and Social Sciences, Royal London House, Bournemouth, BH1 3LT UK; 3 Bournemouth University, Faculty of Health and Social Sciences, Royal London House, Bournemouth, BH1 3LT UK; 4 Service User c/o Orthopaedic Research Institute, Bournemouth University, Faculty of Health and Social Sciences, Executive Business Centre, Bournemouth, BH8 8EB UK; 5 Orthopaedic Research Institute, Bournemouth University, Faculty of Health and Social Sciences, Executive Business Centre, Bournemouth, BH8 8EB UK

**Keywords:** PPI, Patient and public involvement, Impact, Osteoarthritis, Public engagement, Cycling, Exercise, Funding application

## Abstract

**Plain English summary:**

Involving patients and the public in research helps to ensure that research remains relevant, and has an impact on the people it aims to benefit. Funding bodies now require patients and the public to be involved at all stages of research. Patients and members of the public were involved from the outset in research into a cycling and education programme for hip osteoarthritis. A group discussion took place with six participants from a trial of the programme. The group provided feedback on several areas including the relevance of the research, how the researchers proposed to recruit patients, the research design, the programme itself (including what they liked/didn’t like about it), and how the researchers could publicise the research findings. The feedback received was invaluable, and helped shape the entire research project and funding application. The cycling and education programme has been extended in line with comments received from the group. They also helped identify the best way of gathering information from research participants and had suggestions for sharing the results, both of which were incorporated into the funding application. Often involving patients and the public in research can be seen as a ‘tick box’ exercise. However, this example shows how crucial involving patients and the public in research design is. It also shows how the funding application was made stronger as a result of patient input. Researchers should be encouraged to work closely with patients and the public to ensure their research is of the highest quality.

**Abstract:**

**Background**

Involving patients and the public in research is an essential activity to ensure relevant, accessible, and appropriate research. There is increasing obligation from funding bodies on researchers to have well thought through plans for involving the public, and indeed it is often a condition for funding. Patient and public involvement activity in this project was conducted to inform a funding application to investigate the effectiveness of a cycling and education intervention in the treatment of hip osteoarthritis.

**Methods**

Six participants from a feasibility programme of the intervention attended a two-hour patient and public involvement consultation group to provide feedback on various aspects of the proposed research and intervention. During the consultation group, two independent facilitators followed a detailed plan formulated with the research team. Feedback was validated by the attendees via email following the consultation, and a report was issued to the research team. Further feedback on subsequent changes was sought via email and telephone with members of a Patient Advisory Group.

**Results**

The patient and public involvement consultation group provided invaluable feedback and suggestions which impacted on the design and quality of the research project and the intervention. Key changes to the intervention included extending the duration of the cycling programme from six to eight weeks, and inclusion of an exercise diary to promote adherence to the intervention. Key feedback regarding the design of the research and funding application included suggestions for methods of dissemination, and confirmation of the primary outcome measure.

**Conclusions**

Patient and public involvement was crucial to the design of the proposed research and intervention. It informed many aspects of the research design and made the funding application stronger as a result. Involving patients and the public in research is much more than an obligation, or ‘tick box’ exercise. It can change and improve research quality, which is crucial when answering questions that are meaningful and important to patients, and which leads to increased impact. Collaboration with patients and the public should be planned and reported from the conception of a research idea where the impact of such input can be considerable.

## Background

### The need for patient and public involvement

Patient and public involvement (PPI) or public involvement in research is defined as “research being carried out ‘with’ or ‘by’ members of the public rather than ‘to’, ‘about’ or ‘for’ them” ([1]: p.6). It is an essential activity in all stages of the research process to ensure the acceptability, relevance, timeliness, and quality of research [1, 2].

The commitment to and recognition and value of public engagement, as well as the need for training and support has been acknowledged for researchers in the Concordat for Engaging the Public with Research [3]. Indeed, there is an increasing expectation from funding bodies that researchers have conducted PPI activity at the stage of applying for funding, and is often a condition of funding [2]. For example, the National Institute for Health Research (NIHR) Research Design Service PPI handbook notes an expectation that applicants to NIHR funding programmes are dedicated to involving patients and the public in their research [2].

The NIHR suggests that patients and the public should be involved throughout the research cycle [2]. When designing a research study and developing a funding application, patient and public views should be sought to confirm the appropriateness of the research question, inform the recruitment strategy, improve the quality of the plain English summary and inform data collection tools and outcome measures [2]. Consulting the public for their opinion and suggestions when writing a plain English summary often leads to a better quality, easier to understand summary, which funders such as the NIHR and bodies such as INVOLVE consider of major importance [4].

As well as ensuring involvement at all **stages** of research, it is also recognised that there are three **levels or approaches** of patient and public involvement in research: consultation, collaboration or user-led involvement with user-led research arguably the highest and ‘best’ level of involvement [1, 5]. Furthermore, INVOLVE highlights the six values for public involvement in research as respect, support, transparency, responsiveness, diversity and accountability [6]. Although each research project differs in its aims and objectives [6], it is generally expected that all approaches and values are carefully considered and implemented where possible. The benefit of PPI is clear, and completed in the right way, can lead to vital feedback regarding the design of research including the proposed research question, outcome measures, recruitment strategies, and methods of dissemination [1, 7].

### The problem being researched

Over two million people in the UK have osteoarthritis of the hip [8] and it is associated with hip pain, stiffness, and dysfunction during activities of daily living. It is the most common reason for a total hip replacement and in 2012 there were 76,448 primary hip replacements in England, Wales and Northern Ireland [9]. National Institute for Health and Care Excellence guidance [10] recommends that education, exercise (both aerobic and local muscle strengthening), and weight loss (if required) should be the first line of treatment for people with hip osteoarthritis. Cycling in particular is a non-weight bearing and low impact exercise that enables many repetitions of hip joint movement compared with typical interventions such as manual therapy used within conventional physiotherapy. Cycling can therefore increase range of movement, strength and function, and reduce pain [11]. It is an excellent form of aerobic exercise and pain perception is known to decrease following aerobic exercise [12]. The cycling and education intervention encourages participants to self-manage their symptoms and recognises that exercise only has a sustained effect for as long as the person continues to exercise. Participants are encouraged to continue exercising after the programme, and to make lifestyle changes that enable them to manage their condition in a sustainable way. Findings from a feasibility study suggest that the programme has been of benefit [13, 14] with participants continuing to exercise following the programme’s conclusion.

The aim of the PPI activity described below was to inform the design of a research project to investigate the effectiveness of a cycling and education intervention compared to standard physiotherapy for the treatment of hip osteoarthritis in Dorset. The feedback received was also used to inform the application for funding to conduct this research. The PPI activity was designed based on the guidance in the NIHR Research Design Service PPI handbook, INVOLVE guidelines, and examples of good practice [15, 16]. Where appropriate, results are reported based on the Guidance for Reporting Involvement of Patients and Public (GRIPP) checklist [5].

## Methods

A feasibility programme of the cycling and education intervention has been running with support from key stakeholders in local leisure centres in Bournemouth since September 2013, and to date 119 participants in eight groups have completed the programme. Dorset was an ideal location in which to hold the feasibility programme as it has one of the oldest populations in the United Kingdom (25 % of over 65 year olds compared to the national average of 16.4 % [17]) and Bournemouth and Poole Hospitals have some of the highest rates of hip surgery in the country (1907 primary hip replacements in Dorset hospitals in 2013 [18]).

At the time of writing the funding application, only the first three groups had completed the programme. All participants from these three groups (N = 30) were contacted by email and asked to attend a service user consultation. These participants had self-referred to the exercise programme due to experiencing hip pain and problems with hip function. Six participants (five male, one female) attended one two-hour consultation group to provide feedback on all aspects of the proposed research as well as their experiences in the feasibility programme. For convenience, the group was held in the evening at the same location the programme had been held (a local leisure centre), refreshments were provided, and in accordance with INVOLVE guidelines participant expenses (i.e., travel, or carer expenses) for the evening were reimbursed by the NIHR Research Design Service South West. Participants also received a £10 voucher for a supermarket of their choice as a thank you for their time. Ethical approval was not required for this consultation [2, 19].

A plan was drawn up in consultation with the Chief Investigator and founder of the programme, and with the wider research team regarding the type of information required from the consultation session. The plan consisted of the topics of interest and example questions for each topic to prompt discussion. The general topics of enquiry were:

- The design of the project (including the importance of the research question, the research design including potential recruitment issues)
- Intervention development (i.e., appropriateness of the location and usefulness of the educational resources e.g., course booklet, exercise diary etc.)
- Appropriateness of research materials (plain English summary and data collection tools)
- The primary outcome/what was most important to them to measure and which tool was most suitable to use to collect such information
- Methods for dissemination of results
- Anything else they felt was of importance to note

The group session was co-facilitated by two facilitators independent of the feasibility programme (and who were not co-applicants on the funding application). The Chief Investigator was not present. This encouraged the group to be open and honest in their feedback, and to challenge the research team’s ideas. All participants had been emailed a copy of the plain English summary of the project prior to the consultation. Copies of proposed outcome measures were also made available to the group on the day. Using the agreed topic guide to stimulate informal discussion, the main points raised were noted on a flip chart. This enabled the facilitators to check that they fully understood the feedback they were being given by the group whilst allowing validation of the information. It also acted as a useful way of collecting consultation data as the session was not audio recorded. Hand-written notes were also taken by each facilitator.

After the session, the facilitators collated the information they had collected (notes and flip chart recordings) and noted the main points raised by the consultation group. A report of the consultation group data was produced and a summary emailed to the participants of the consultation group for information and further validation. They were also asked if they would be interested in assisting throughout the research project, for example as a co-applicant or steering group member. Once further feedback had been received, the report was amended and sent to the research team for their consideration. The facilitators also met with the Chief Investigator to discuss the findings in further detail. This allowed the facilitators to act as a channel through which to exchange information between the consultation group and research team.

Due to a subsequent proposed change in the main outcome of interest, further feedback was sought from a Patient Advisory Group formed of three participants who had expressed an interest in being involved throughout the research project. The Patient Advisory Group were contacted by email initially, and asked to offer their opinion via email or telephone. They were asked to comment on the change of primary outcome, and proposed outcome measure, specifically whether they agreed with the change of primary outcome and how the alternative measure compared to the measure they had reviewed in the consultation group. At this stage, the Patient Advisory Group was also asked to review the plain English summary that had evolved in line with comments from the consultation group, and the proposed methods of recruitment that had not been covered in the consultation group due to lack of time. One participant requested a discussion over the telephone, and the other two provided feedback by email. A summary of this feedback was sent by email to the research team as before.

## Results

Overall, the group gave positive feedback about the proposed research. They agreed the plain English summary was clear and easy to understand, and agreed that the research question was appropriate. The feedback provided by the consultation group and through further discussion via email and telephone was considered by the Chief Investigator and wider research team, and changes to the design and intervention were discussed and made as appropriate. The main points and how they were addressed are summarised below (see Table 1 for a breakdown of outcomes and impacts):

**Table 1:** The outcomes and impacts of feedback from a patient and public involvement consultation group

Theme	Feedback received (outcomes)	Action taken (impacts)
Design	Participants were concerned about randomised controlled trial design	Methodological importance of randomised controlled trial design will be highlighted in Patient Information Sheet
	Inclusion of a qualitative component suggested	This was initially incorporated into the research design but was later removed in favour of an economic evaluation due to funding constraints
Intervention	Programme not long enough	Extended from six to eight weeks
	Leisure centre location was ideal	Will remain in leisure centre
	More time for questions/group discussion required	Incorporated into intervention
	Further supplementary material required	Incorporated into intervention
	Exercise diary was useful and motivational	Confirmed inclusion in intervention
Research materials	Data collection tools deemed appropriate and not burdensome	The same data collection tools will be used in the proposed study
	A questionnaire for self-efficacy unnecessary	Was not included
Main outcome of interest	Pain/Oxford Hip Score to be primary outcome and later confirmation of Western Ontario and McMaster Universities Osteoarthritis Index (WOMAC)	Due to being considered a higher quality measure than the Oxford Hip Score in a recent Cochrane review [23], WOMAC was chosen as the primary outcome. Its appropriateness was confirmed by the Patient Advisory Group.
Dissemination	Suggested General Practitioners, scientific journals, media including radio important	Have been incorporated into dissemination plan

Feedback received from a patient and public involvement consultation group in Bournemouth, 2014 and how it has been incorporated into the design of a research project and funding application to evaluate a cycling and education intervention in the treatment of hip osteoarthritis

### Design of project

*As well as their general observations about the project, the group’s specific feedback about the importance of the research question, their views about the research design (randomised controlled trial) and their feelings about including a qualitative component were also sought.*

#### Outcomes

When discussing the research design, the group did not initially support the idea of a randomised controlled trial as the randomisation process means there would be a chance that participants would not receive the intervention. They also felt that a crossover design (i.e., having both treatments in turn) would not be appropriate as the programme is based upon on-going self-management and continuing to exercise is crucial. The group supported the incorporation of a qualitative component but suggested that telephone interviews rather than face to face interviews might be more convenient and preferable for some participants. The group confirmed that they would be happy to report information on General Practitioner (GP) visits and use of pain relief medication for the evaluation of cost-effectiveness.

#### Impact

The consultees’ concerns regarding the standard randomised controlled trial design may well have been magnified given they had already completed the programme and perceived to have benefitted from it. Their concerns were originally shared by the research team. However, the researchers felt that a randomised controlled trial would be the most appropriate and most rigorous design to be able to answer the clinical question. The importance of the randomised controlled trial design to determine best practice will therefore be highlighted in the Patient Information Sheet and explained to participants.

The researchers agreed with the consultation group, and recognised the importance and benefit of including a qualitative component and had planned to include telephone interviews as suggested. However, after careful consideration a cost-effectiveness analysis was included in preference to a qualitative component in the submitted grant application. This decision was made entirely due to the constraints in research costs from the target funder. It was also anticipated that policy makers would find results from a cost-effectiveness analysis more useful when deciding whether to introduce the intervention into practice. Although the research team had hoped to incorporate a qualitative component to the research, the data collection tools to be used in the proposed study are patient reported outcome measures and so important patient views will still be captured.

### Feedback on the programme/intervention development

*As well as general feedback about the exercise intervention itself, specific feedback about the environment, the equipment, the venue and the education sessions themselves was sought.*

#### Outcomes

Patients liked the cycling class but felt that the programme was not long enough. They therefore suggested extending the course from six to eight weeks with the last two weeks spent concentrating on the practical aspects rather than the theory. The group agreed that holding the course in a leisure centre was both ideal and convenient, and in some cases encouraged them to do more classes.

They found that supplementary information (including videos, emails, material about stretching exercises and links to educational websites etc.) was very helpful. However, they would have liked more time for questions and opportunity to talk between the education session and the cycling session. Furthermore, development of the programme over time meant that some participants had been given an exercise diary to complete whereas other participants had not. Those who had received a diary had found it useful to keep on track and to see their progress. They felt it helped develop good exercise habits and provided a motivation to exercise.

A member of the Patient Advisory Group and co-author of this paper stated:

*“I’ve had this problem for 46 years and surgery seemed like the only option until starting this course. I’m still cycling every day and it helps to lubricate my hip.”*

#### Impact

On the basis of the group suggestions alone and despite the additional associated treatment costs, the intervention was extended from six to eight weeks with the last two sessions concentrating on consolidating cycling technique rather than the educational sessions. Providing supplementary information (i.e., on stretching exercises) between sessions has also been incorporated into the programme. The programme will remain based within leisure centres as the group found this to be most appropriate, as opposed to being based in an outpatient physiotherapy department which may have been more pragmatic in terms of recruitment and future commissioning potential. The research team hadn’t previously appreciated the value participants placed on the discussion time in the education sessions, and so more time for questions has been incorporated. An exercise diary will now be universally provided to increase motivation and adherence to the intervention.

### Research materials

*Participants were asked how they felt about the proposed data collection tools to be used in the subsequent research.*

#### Outcomes

The group were happy with the format of the data collection booklet and the provision of personal data required for completion. They felt that the time taken to complete them would be acceptable and would not be a burden on participants. The research team considered whether self-efficacy (people’s beliefs and judgements about their ability to reach goals [20]) should be explored as part of the study but the group felt this was unnecessary.

#### Impacts

Despite being considered important by the research team, as the need to investigate self-efficacy was deemed unnecessary by the consultation group this has not been included in the research design. However, on reflection perhaps this concept was not fully explained to the group. Had this been discussed further it is possible that the consultees would have considered it important to include a measure of self-efficacy.

### Main outcome of interest

*Participants’ views about the most appropriate primary outcome were sought.*

#### Outcomes

The group agreed that they had all signed up to the programme to decrease the levels of pain in their hip (and avoid surgery) and that pain should be the main outcome of interest for the proposed study. They agreed that the Oxford Hip Score [21] captured the most important responses well, and would be the best measure to use as this measure had been used throughout the feasibility programme.

#### Impacts

The research team agreed that the Oxford Hip Score [21] was the most appropriate measure to use as the primary outcome. However, after a further detailed literature review and subsequent analysis of results from the feasibility programme [14], the Western Ontario and McMaster Universities Osteoarthritis Index (WOMAC) [22] was found to be a more sensitive measure than the Oxford Hip Score. Furthermore the WOMAC was used in seven out of the ten studies included in a recent Cochrane Review [23] and was considered a higher quality measure than the Oxford Hip Score in a hierarchy of outcomes used to extract data in the review. Despite the group’s suggestion that pain should be the primary outcome, it is now proposed to use the physical function score of the WOMAC as the primary outcome. Although patients can focus on pain as the problem, when they become more active (e.g., by cycling), their functional scores improve and subsequently, their pain levels also improve [14]. This proposed change was discussed with the three members of the Patient Advisory Group. All three supported the change, agreeing that the WOMAC was easy to complete and captured the most relevant information regarding both function and pain.

### Dissemination

*Participants were asked for their preferred methods of dissemination, and key target audiences.*

#### Outcomes

The group had several suggestions regarding the dissemination avenues that could be utilised in this project. The group agreed that the most appropriate place to disseminate the information would be in GP surgery waiting rooms. They felt that disseminating information on blogs, or Twitter would be less useful but recognised the importance of publishing in scientific journals to reach clinicians. One participant suggested disseminating through the local authority health centres or liaising with them to produce bike hire offers, or leisure centre offers etc. It was also suggested that one method of dissemination could be via the radio – specifically the ‘Inside Health’ programme with Dr. Mark Porter on BBC Radio 4.

#### Impacts

The group raised many points that the research team had not considered and so the dissemination and impact plan was amended in accordance with feedback received. Flyers, posters and leaflets will be distributed to GP surgeries for display in waiting rooms. Results will also be discussed with key stakeholders such as GPs to incorporate into their clinical practice and discuss with appropriate patients. Despite the group’s feeling that using social media would be less useful, information will be disseminated via Twitter, blogs, and websites to target the public but also clinicians and academics with an interest in the field. Information will also be disseminated through press releases e.g., local, regional and national newspapers, freelance health reporters and radio, as suggested by the group.

## Discussion

For the purposes of this activity, PPI was defined as the active involvement of patients in the designing of a research project to evaluate a cycling and education intervention for the treatment of hip osteoarthritis. Good models of practice for involving the public at an early stage in the research process were followed [2, 15, 16]. Involvement at the funding application stage was included at consultation level with the view to involve patients and the public at a collaborative level throughout the delivery of the research (and in the development of this paper). The views of the consultation group were sought on the appropriateness of the research question, recruitment strategy, data collection tools, plain English summary and outcomes of interest [2].

Despite the limitations discussed below, the feedback obtained from the consultation group influenced many aspects of the research design of the proposed randomised controlled trial (see Table 1). Often, PPI is perceived as a ‘tick box’ exercise, especially by busy clinicians and academics who may not fully understand its value. More worryingly, researchers can often confuse PPI with research participation rather than understanding that the two are separate [2]. However, it is often only through conducting PPI activity that a fuller understanding of its value is achieved. The Chief Investigator has now experienced first-hand the importance of PPI, and states:

*“The PPI activity proved insightful to the process of developing the research proposal. I was delighted and surprised with the level of engagement that the participants provided. The outputs were of high value and stimulated discussion and debate around research design issues that we had not previously considered. The PPI feedback, when used in combination with the findings of our feasibility work undoubtedly helped us to improve the methodological design of our study.”*

The feedback provided by the consultation group proved invaluable and was incorporated where possible when designing the study and preparing the funding application. It helped to improve the quality of the research by ensuring the research is patient-centred whilst remaining methodologically sound. It also ensured the intervention is appropriate and provided suggestions to improve the intervention and increase its effectiveness. PPI is a vital step in the research design process. Done in the right way, it has the potential to lead to a better research design [1, 7] which is more likely to lead to better outcomes that have more impact. A commitment from research teams is therefore crucial to ensure PPI activity is taken seriously rather than being tokenistic.

Although the research team had hoped to incorporate and address as much of the group feedback as possible, there were aspects that could not be for the reasons described above (i.e., inclusion of a qualitative component and choice of primary outcome measure). Suggestions were only not incorporated where they compromised methodological rigour or due to funding constraints outside the research team’s control. The change in primary outcome measure arose as a result of the feasibility project evolving and further literature searching highlighting a more appropriate measure to use. Despite copies of the proposed outcome measures being made available in the consultation session, the WOMAC was not included in these measures. The research team should have considered and discussed other primary outcome options and data collection tools earlier to present to the consultees for their feedback, which may have highlighted the need to change the primary outcome earlier. Although important to involve patients and the public at as early a stage as possible [2], planning is also important to ensure comprehensive feedback is obtained without the need to repeat PPI activity at the inconvenience of patients and the public. The research team now recognise the importance of planning, and recommend that it is carefully considered when conducting PPI activities. Three participants from the consultation group volunteered to form a collaborative Patient Advisory Group for the future study and so the research team were able to contact the group for more feedback when required, particularly to discuss changes to the primary outcome measure. Indeed, a member of the Patient Advisory Group has collaborated in writing this article and is a co-author on this paper. Of his involvement he states:

*“People with the problem are the most able to help researchers solve the problem for others. Patient involvement is absolutely crucial at all stages of research, but it is important to involve them in a way that is acceptable or suitable to them, i.e., keep it simple. I think the team have done a huge amount of work and I have acted as a sounding board. The researchers have made it very easy to take part.”*

Further limitations of the activity described in this paper include the limited time for discussion in the consultation group. The meeting lasted for two hours, which proved insufficient to capture all the participants’ views. However, the research team did try to address this limitation by welcoming further follow-up feedback via email after time for reflection, and involving members of the Patient Advisory Group for their feedback on aspects of the research design that had not been discussed. Also, holding similar sessions with participants from the other groups of the feasibility programme may have provided more diverse feedback. Furthermore, of those six participants who attended, only one participant was female in contrast to the feasibility programme where more females attended. Whilst holding further groups may have helped to address this imbalance, due to the self-selecting nature of PPI it is widely acknowledged that PPI activity often attracts a different socio-demographic group.

As described above, the facilitators of the consultation group were independent of the feasibility study and were not co-applicants on the funding application, meaning that the consultees did not have any direct contact with the research team. This was to ensure that the consultees felt they could be open and honest, and that challenging the researchers’ ideas was welcome. The participants had all previously met the Chief Investigator as the feasibility programme lead, and therefore it was felt that involving him in the consultation may have influenced patient responses. However, on reflection the research team would recommend that the findings from the initial group were discussed with a subsequent group, taking a more collaborative approach and providing an opportunity for the Chief Investigator to become more involved in the PPI process. Furthermore, it would have allowed for direct dialogue between the research team and the consultees rather than using facilitators to channel information as was the case in this example. The consultees received a summary of the discussion and the main points they raised, and were informed of the changes made to the research design. However, decisions made by the research team as a result of the consultees’ points should have been fed back to the group more promptly and even confirmed with further groups. The researchers recommend that feedback is always provided promptly to PPI participants to inform them of the impact they have had on a research design.

Although involvement at a consultation level is important, collaboration is typically considered to add more value and user-led involvement even more so, as research ideas come directly from the users and consequently will likely create more impact. However, user-led involvement may not be suitable for all types of research, for example where clinical issues are highlighted in practice. Nevertheless, involvement using collaborative or user-led approaches should be implemented and encouraged wherever possible both prior to funding and after funding is awarded. Indeed, the recent report from the ‘Breaking Boundaries’ review of public involvement in NIHR research includes an aim to include the ‘public as partners’ in all NIHR research [24]. Throughout the proposed research the Patient Advisory Group will be fully involved in the collaborative delivery of the research as suggested by the NIHR [2], including in the design and management of the research, developing participant information resources, undertaking the research, analysing and interpreting the findings, contributing to the study reports, and dissemination activity. The six core values of involving the public in research as suggested by INVOLVE [6] will be followed.

The PPI handbook produced by the NIHR Research Design Service [2] is an extremely useful resource when planning PPI activity, and INVOLVE has a wealth of guidance for researchers in engaging the public in research [1, 6, 25]. Evans et al. [15] have produced guidance for researchers in the form of a standard operating procedure for involving service users in research, and examples of good practice have been published [16] but further work is required. When reporting the findings from this PPI activity, the authors consulted the GRIPP checklist [5]. However, this checklist is largely based on how to report the outcomes and impact of PPI activity once the research is completed and when impact can be more accurately reported. The GRIPP 2 [26] is in development and will include two versions; one version will provide guidance for papers focussing on PPI activity, and the other for papers focusing on reporting study findings. There is a clear need for further high quality reporting of PPI activity [5] and so further guidance around how to report PPI activity prior to funding, and ahead of conducting research would also be of benefit. Models of good practice and guidelines regarding PPI will be followed throughout the proposed randomised controlled trial. When reporting the findings, the GRIPP checklist (and GRIPP 2 when available) will be followed and will include economic appraisal and descriptions of measuring and capturing impacts of the PPI activity [5].

## Conclusions

Including patients in designing the proposed study was of great benefit and an integral part of the funding application - not just a ‘tick box’ exercise. Their views informed and changed many aspects of the research design including the intervention, data collection, primary outcome measure, and methods of dissemination which improved the quality of the research and allowed the research team to submit a much stronger application for funding. However, it also demonstrated the need for a more collaborative approach and more planned involvement from the outset. Therefore, researchers should be encouraged to plan and to collaborate with patients and the public as early as possible as well as feedback on the impact their input has had on the research design. Similarly, service users should be encouraged to come up with research ideas to take forward with clinicians and academics in order to ensure that research directly benefits users. Although commitment to PPI activity and reporting is increasing, more publication and reporting guidelines for pre-funding PPI activity are called for to further embed PPI as essential good research practice.

## Abbreviations

GP: General Practitioner
GRIPP: Guidance for Reporting Involvement of Patients and Public
NIHR: The National Institute for Health Research
PPI: Patient and public involvement
WOMAC: Western Ontario and McMaster Universities Osteoarthritis Index
